# Sugars Increase Non-Heme Iron Bioavailability in Human Epithelial Intestinal and Liver Cells

**DOI:** 10.1371/journal.pone.0083031

**Published:** 2013-12-10

**Authors:** Tatiana Christides, Paul Sharp

**Affiliations:** 1 University of Greenwich, Faculty of Engineering & Science, Department of Life & Sports Science, Chatham Maritime, United Kingdom; 2 King’s College London, Diabetes & Nutritional Sciences Division, School of Medicine, London, United Kingdom; University of Florida, United States of America

## Abstract

Previous studies have suggested that sugars enhance iron bioavailability, possibly through either chelation or altering the oxidation state of the metal, however, results have been inconclusive. Sugar intake in the last 20 years has increased dramatically, and iron status disorders are significant public health problems worldwide; therefore understanding the nutritional implications of iron-sugar interactions is particularly relevant. In this study we measured the effects of sugars on non-heme iron bioavailability in human intestinal Caco-2 cells and HepG2 hepatoma cells using ferritin formation as a surrogate marker for iron uptake. The effect of sugars on iron oxidation state was examined by measuring ferrous iron formation in different sugar-iron solutions with a ferrozine-based assay. Fructose significantly increased iron-induced ferritin formation in both Caco-2 and HepG2 cells. In addition, high-fructose corn syrup (HFCS-55) increased Caco-2 cell iron-induced ferritin; these effects were negated by the addition of either tannic acid or phytic acid. Fructose combined with FeCl_3_ increased ferrozine-chelatable ferrous iron levels by approximately 300%. In conclusion, fructose increases iron bioavailability in human intestinal Caco-2 and HepG2 cells. Given the large amount of simple and rapidly digestible sugars in the modern diet their effects on iron bioavailability may have important patho-physiological consequences. Further studies are warranted to characterize these interactions.

## Introduction

Evidence that simple sugars such as glucose and fructose affect iron bioavailability first arose in the 1960s from work showing that sugars were able to chelate inorganic iron and form stable, low molecular weight soluble complexes [1]. These sugar-iron complexes were readily absorbed across the intestinal mucosa of rodent models [2], [3]. Given that intake of fructose and sucrose has increased dramatically worldwide in the past 40 years, especially in the Western world, while at the same time iron deficiency and iron excess remain significant public health concerns [4]–[6], understanding the nutritional implications of iron-sugar interactions is particularly relevant.

Excess sugar is blamed for a myriad of modern health problems, but whether sugars might actually be protective against iron deficiency, or contribute to either total body or cellular iron overload is unknown. Insufficient body iron levels are associated with significant health consequences, and approximately 2 billion people suffer from iron deficiency. Furthermore, iron overload related to either primary (e.g. hereditary hemochromatosis) or secondary (e.g. beta-thalassemia) abnormalities in iron metabolism is prevalent in many populations [6], [7]. There is also interest in the role that disordered regulation of intracellular iron levels plays in the pathogenesis of several non-communicable diseases including non-alcoholic fatty liver disease (NAFLD) [8], [9].

Absorption of non-heme iron begins with iron uptake into the enterocyte by Divalent Metal Transporter 1 (DMT1); DMT1 takes up ferrous iron (Fe^2+^) ([10], [11], reviewed by Montalbetti et al, [12]). Non-heme iron, however, is primarily in the oxidized ferric form (Fe^3+^) thus it must be reduced to be absorbed; reduction occurs either via the apical membrane bound ferrireductase Duodenal cytochrome b (Dcytb), or through reducing agents such as ascorbate [13]. Dietary factors can change non-heme iron bioavailability by altering iron solubility or oxidation state.

Non-heme iron is the main source of iron in the diet [14] and its bioavailability is influenced by a range of dietary factors. Studies investigating the influence of sugars on iron bioavailability have yielded conflicting results; although a number of studies demonstrated improved iron bioavailability [15]–[20], others found either no effect [21], [22], or decreased absorption [23], [24]. Human studies have been few, small (number of subjects ranged from 8–25 in the above cited studies), of short duration [17], [23], and with limited information on iron status and hereditary iron metabolism defects [15], [22], [23].

The most consistent finding regarding mono- and di-saccharides and iron is that fructose increases dietary non-heme iron absorption, possibly by chelating and/or reducing iron to the ferrous form [25]. Whilst the dietary burden of fructose alone is low, consumption of sucrose (a glucose-fructose disaccharide cleaved into its constituent sugars prior to absorption), and high fructose corn syrup (HFCS, a widely used liquid sweetener), is high [26]–[28]; consequently fructose levels in both the gut and portal vein may be elevated. It is thus vital to clarify the effect of sugars and sweeteners on iron bioavailability in the gut and liver, as this could have an impact on iron status, particularly in population groups at risk of iron overload.

The objective of the current study was to investigate the effects of the sugars fructose, glucose and sucrose, as well as high fructose corn syrup 55 (HFCS-55, a mixture of fructose and glucose monomers in a 55:45 ratio), on non-heme iron bioavailability using the Caco-2 cell in vitro digestion model. Furthermore, as the modern diet delivers a loaded cocktail of sugars and iron to the liver, and fructose may be used in studies to induce hepatic metabolic changes, the effect of sugars on liver iron absorption was evaluated using the liver-derived HepG2 cell line. The in vitro digestion Caco-2 cell model is an established tool for assessing gut iron bioavailability and has been validated by comparison with data from human studies [29], and HepG2 cells have been used to measure liver iron uptake [30]. Ferritin formation in both cell lines correlates with increasing concentration of iron treatments [31]–[33]. The use of ferritin as an indicator of iron availability was pioneered by Glahn et al, [34] and it is now widely used as a surrogate marker for iron uptake (for recent examples see [35]–[38]).

## Materials and Methods

### Reagents

Unless otherwise stated, reagents were purchased from Sigma-Aldrich, UK. Glassware used in sample preparation and analyses was treated with 10% (v/v) concentrated Nitric acid (68%) for 24 h and rinsed with 18 MΩ purity water. All water used in experiments was18 MΩ purity.

### Cell culture

The Caco-2 TC7 cell clone, developed by Monique Rousset and colleagues [39], [40], was kindly gifted to the Sharp lab and was used in experiments at passages 44 – 49. Cells were maintained in cell culture treated T75 flasks (Corning Inc., Costar) and subcultured every 5–7 days. Cells were grown in Dulbecco’s Modified Eagle Medium (DMEM, Gibco, 41965) supplemented with 10% v/v fetal bovine serum (LCG Standards, 30-2020), 1% penicillin-streptomycin, 4 mmol/L L-glutamine, 1% non-essential amino acids, and Plasmocin 5 µg/ml (Source Bioscience). For experiments, Caco-2 cells were seeded at 1×10^4^ cells/cm^2^ in six-well plates (Corning Inc., Costar) and used 13-15 days post seeding as per the protocol used in the Glahn lab [34]; at this stage DMT1 protein levels and iron uptake are maximal in the Caco-2 TC7 cell line [41].

HepG2 cells were obtained at passage 28 from American Type Culture Collection and used in experiments at passages 30–40; cells were maintained in cell culture treated T75 Tissue Culture Flasks seeded at a density of 1×10^5^ cells/cm^2^ and sub-cultured every 48 – 72 hours. Cells were grown in DMEM supplemented with heat inactivated 10% v/v fetal bovine serum, 1% penicillin-streptomycin, 2 mmol/L glutamine, 1% non-essential amino acids. Experiments with HepG2 cells were carried out in cell culture treated six-well plates seeded at 1×10^5^ cells/cm^2^ and used 24- 48h post seeding.

24 hours prior to all experiments (Caco-2 & HepG2) DMEM medium was removed and the cell culture wells washed with 2.0 ml Minimal Essential Medium (MEM, Gibco, 31095); growth medium was then changed to MEM supplemented with 10 mmol/L PIPES (piperazine-N, N’-bis- [2-ethanesulfonic acid]), 1% antibiotic/ antimycotic solution, 11 µmol/L hydrocortisone, 0.87 µmol/L insulin, 0.02 µmol/L sodium selenite (Na_2_SeO_3_), 0.05 µmol/Ltriiodothyronine and 20 µg/L epidermal growth factor. Fetal bovine serum (FBS) free media was used because different batches of FBS have differing levels of iron and other factors that could add confounding variables; MEM was supplemented to ensure optimal Caco-2 cell growth and differentiation in the absence of FBS while maintaining iron levels < 8 mg Fe/L [34] [42].

### Caco-2 cell - in vitro digestion studies

**Sugar solutions.** All solutions were freshly made on the day of the experiment. Stock solutions of 1 mol/L fructose, glucose or sucrose were prepared in 140 mmol/L NaCl, 5 mmol/L KCl, pH 2 solutions. In addition, HFCS-55 (a kind gift from Hanseland, Groningen, Holland) was diluted with water to produce a 1 mol/L fructose stock solution and then shaken with 4 g Chelex 100 resin (Bio-Rad Laboratories, 142-2832) for half an hour to remove possible metal contaminants, followed by elution through a 1.6 cm diameter filtration column (VWR). Iron levels in the Chelex treated HFCS stock solutions were checked by Inductivity Coupled Plasma-Optical Emission Spectrometer and were < 5 µmol/L; iron levels in blank, no food digests were also < 5 µmol/L. Sugar concentrations were selected to be within the range that might occur in the gut after a meal (after dilution through the in vitro digestion). All solutions were filter sterilized prior to cell culture application.

The in vitro digestion followed a modified version of the protocol developed by Glahn et al [43]. All digestion solutions were prepared fresh for each experiment. On the first day of the experiment the cells were washed with 2.0 ml MEM and 1.0 ml supplemented MEM was added to each individual plate well.

Food samples were prepared as follows: 25 µg of Fe (added as Fe solubilized in 1% HCL, High-Purity Standards, 100026-2) and 1.0 mL of stock sugar solutions were added to 10 ml 140 mmol/L NaCl, 5 mmol/L KCl pH 2 solutions in sterile 50 ml polypropylene centrifuge tubes. The ratio of iron:sugar ≈ 1∶2000 was based on expected relative values of the two nutrients in the gut. Reference control samples of 25 µg Fe added to 11 ml 140 mmol/L NaCl, 5 mmol/L KCl, pH 2 solutions alone, as well as positive controls consisting of 25 µg Fe added to 11 ml 140 mmol/L NaCl, 5 mmol/L KCl, pH 2 solutions containing 265 µmol/L ascorbate were performed with each replication. The positive controls’ iron:ascorbate ratio was chosen to reflect typical relative levels that might occur in a meal. To ensure no iron contamination of the system “no food digest” samples consisting simply of 140 mmol/L NaCl, 5 mmol/L KCl, pH 2 solutions were included with each experiment. Finally, solutions of 140 mmol/L NaCl, 5 mmol/L KCl, pH 2 containing 1 ml of stock sugar solutions without extraneous iron were tested to ensure that sugars alone did not increase ferritin formation. In some experiments, known inhibitors of iron bioavailability, phytic acid and tannic acid, were added to the food digests.

The peptic phase of digestion was initiated by the addition of 0.5 ml pepsin solution (Chelex purified) to each food sample (herein referred to as digests or food digests). The pH was readjusted to pH 2.0 with 1 mol/L HCL and the samples were shaken in a New Brunswick Orbital shaker at 37**°**C, 200 RPM, for 75 minutes. This phase was terminated with the addition of 1 mol/L NaHCO_3_ and subsequent pH increase to ∼ pH 5.5.

The intestinal digestion phase was initiated with the addition of 2.5 ml Chelex-purified bile/pancreatin solution with subsequent adjustment of the pH to pH 6.9 – 7.0 with 1 mol/L NaHCO_3_. All food digests were then brought to a final volume of 15 ml by the addition of 140 mmol/L NaCl, 5 mmol/L KCl solution, pH 6.9.

1.5 ml aliquots of digests were gently pipetted into the upper chamber of each cell culture plate well; the upper chamber was created by the fitting of a 15,000 Da molecular weight cutoff dialysis membrane (Tubing Spectra/Por 7 dialysis membrane, Fisher Scientific) to a Transwell tissue culture treated insert ring (Fisher Scientific; the necks of the rings were shortened by 0.1 mm to remove the original filter and prevent excessive pressure on the cell monolayer) held in place with a silicone ring (Parker 2-023 S0613, WebSeal Inc.). Plates were then covered and placed on a platform fitted Multi-function 3D rotator (Fisher Scientific PS-M3D) set at 6 oscillations per minute in a 37**°**C incubator with a 5% CO_2_/95% air atmosphere at constant humidity for 120 minutes.

After the 120 min incubation the inserts were removed and an additional 1.0 ml of supplemented MEM was added to each cell culture plate well. Plates were returned to the incubator for a further 22 hours; after this period cells were harvested for analysis of cell ferritin content.

Six replicates of each sugar were tested per experiment, and each experiment was repeated at 3 separate times.

**Fructose Analysis.** Levels of fructose in prepared fructose solutions and HFCS-55, before and after Chelex treatment, were determined using high performance liquid chromatography (HPLC) with refractive index detection using water/methylated spirit extraction method with modifications for high salt levels. Premier Analytical Services, accredited by the United Kingdom Accreditation Service (UKAS), Buckinghamshire, UK, carried out the analysis.

### Measurement of iron in blank no-food-digests and HFCS-55 by ICP-OES

Aliquots of the HFCS-55 Chelex treated stock solutions and blank digests were subjected to microwave digestion using an accelerated reaction system (CEM MARS 5**®** with XP-1500 vessels). 0.5 ml of the solutions (in triplicate) was added to 5.0 ml concentrated 68% trace analysis grade nitric acid (Fisher). The samples were heated for 20 minutes at 400-psi pressure and 1200-W power. Iron levels were quantitatively analyzed by Inductivity Coupled Plasma-Optical Emission Spectrometer (ICP-OES, Perkin Elmer Optima 4300 DV).

### HepG2 cell studies

HepG2 cells were treated for 24 h with MEM containing 1 µmol/L ferric ammonium citrate (FAC) and either 15 mmol/L glucose (inclusive of the 5 mmol/L glucose contained in MEM) or 1 – 15 mmol/L fructose. Cells treated with MEM with 1 µmol/L FAC alone served as a reference in each experiment; in addition, 1 µmol/L FAC + 100 µmol/L ascorbate, and MEM alone treated cells were positive and negative controls, respectively. HepG2 cells were also treated with fructose in the absence of any added iron to ensure that fructose alone did not increase ferritin formation unrelated to iron uptake. After 24 hours cells were harvested for analysis of cell ferritin content.

### Ferritin analysis

At the end of each experiment, medium was removed from the wells and cells were rinsed twice with ice cold Phosphate Buffered Saline (PBS). 200 µl ice cold CelLytic with 1% protease inhibitor was added to each well, and cell monolayers were removed with a cell scraper and placed in 1.8 ml Eppendorf tubes. Tubes were shaken for 15 minutes on a Stuart microtitre plate shaker at 1250 RPM and then spun at 6,000 g for 6 minutes in a 5804R Eppendorf centrifuge. The supernatant was aspirated and stored at –80**°**C until analysis.

Ferritin analysis using SpectroFerritin MT Enzyme Linked Immunoassay (ELISA; RAMCO) was carried out on cell extraction supernatants. Absorption readings were performed at 492 nm with subtraction for background at 620 nm in a Thermo Multiscan Ascent Spectrophotometer.

Protein concentration in each sample was measured using the Pierce Protein BCA Assay (Fisher Scientific, 23227). Using this method protein concentrations were consistently 5.0–6.5 mg/ml or 1.0–1.3 mg/well; these are the levels typically found in our lab on day 14 with an initial Caco-2 cell seeding density of 1×10^4 ^cells/cm^2^ (as recommended by ATCC).

### Effect of sugars on iron reduction in vitro

The ferrozine assay is generally used to measure ferrous iron levels in biological samples, and has specifically been used to measure iron in cell extracts [44]. It may also, in adapted form, be used to measure ferrous iron in non-biological samples such as intravenous iron sucrose solutions (Venefor) [45]. In our studies the ferrozine assay was adapted to measure ferrous iron formation in solution after incubation of ferric chloride with fructose, or glucose, or sucrose. The lower limit of detection of the assay is 10 µmol/L; to ensure that production of Fe^2+^ was within the detection limits of the assay we increased levels of iron and sugar in the test tube by a factor of 10 (i.e. 100 µmol/L FeCl_3_; 500 mmol/L sugar) while maintaining the same molar ratio (1∶50). Carbohydrate solutions containing 500 mmol/L glucose, fructose, or sucrose were prepared in PBS; ferric chloride was added to give a final concentration of 100 µmol/L Fe^3+^. 150 µl ferrozine reagents (containing 32 mg ferrozine, 32 mg neocuprine and 3.8 g ammonium acetate dissolved in 10 ml water) were mixed with 400 µl carbohydrate solutions. The samples were incubated for two hours at 37**°**C degrees in a 96 well culture plate. The standard calibration curve was made up with freshly prepared ferrous ammonium sulfate. Absorption readings were performed at 550 nm on a Bio-TekSynergy HT Spectrophotometer.

### Statistical analysis

Statistical analysis of the data was performed using GraphPad Prism (v.6.0 GraphPad Software, San Diego, CA). Statistical analysis was conducted according to the methods of Motulsky [46]. Where noted data from separate experiments were normalized to the relevant reference control. To compensate for unequal variance, GraphPad Prism was used to log transform data. Data are presented as means ± S.E.M. Except as otherwise noted, data were analyzed by one-way ANOVA followed by Tukey’s post-hoc test for pairwise comparisons of experimental groups. Differences between means were considered significant at p ≤ 0.010.

## Results

### Effect of sugars on cell ferritin formation

**Figure 1:**
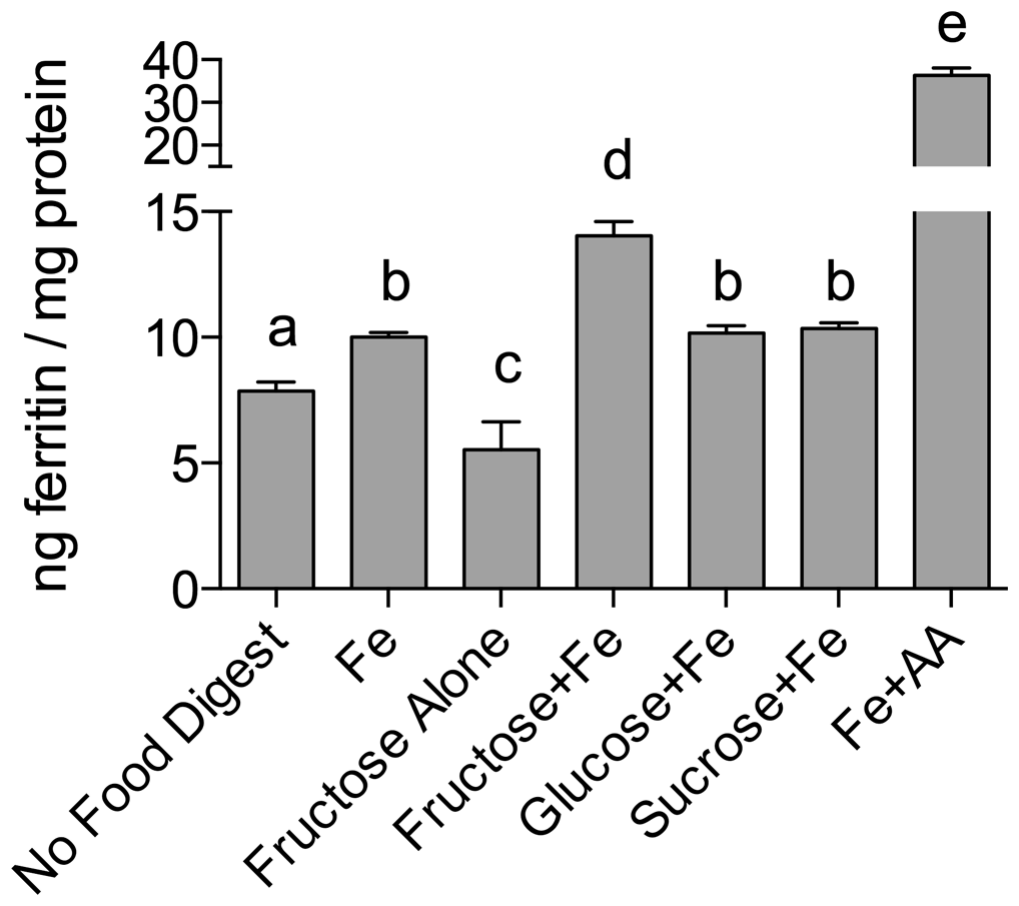
Sugar digests effects on iron-induced ferritin. Measurement of Caco-2 cell ferritin formation from digests of Fe and solutions of sucrose (Sucrose+Fe) or glucose (Glucose+Fe) or fructose (Fructose+Fe) at an iron:sugar ratio of ≈ 1:2000. Equal amounts of iron (25 µg) were combined with sugar solutions (1.0 mL) and subjected to the Caco-2 in vitro digestion process. Digests with fructose alone and no added Fe (No Food Digest) were used as negative controls; digests with Fe alone (Fe) and Fe plus ascorbic acid (Fe + AA) were used as reference controls and positive controls, respectively. Values are means of data normalized to 10 ng of ferritin/mg protein in the reference control (Fe) ± SEM, n≥15. Based on an ANOVA (p<0.0001) with Tukey’s multiple comparisons test post-hoc analysis done on an all-pairwise basis, bar values with no letters in common are significantly different (p ≤ 0.010).

**Figure 2:**
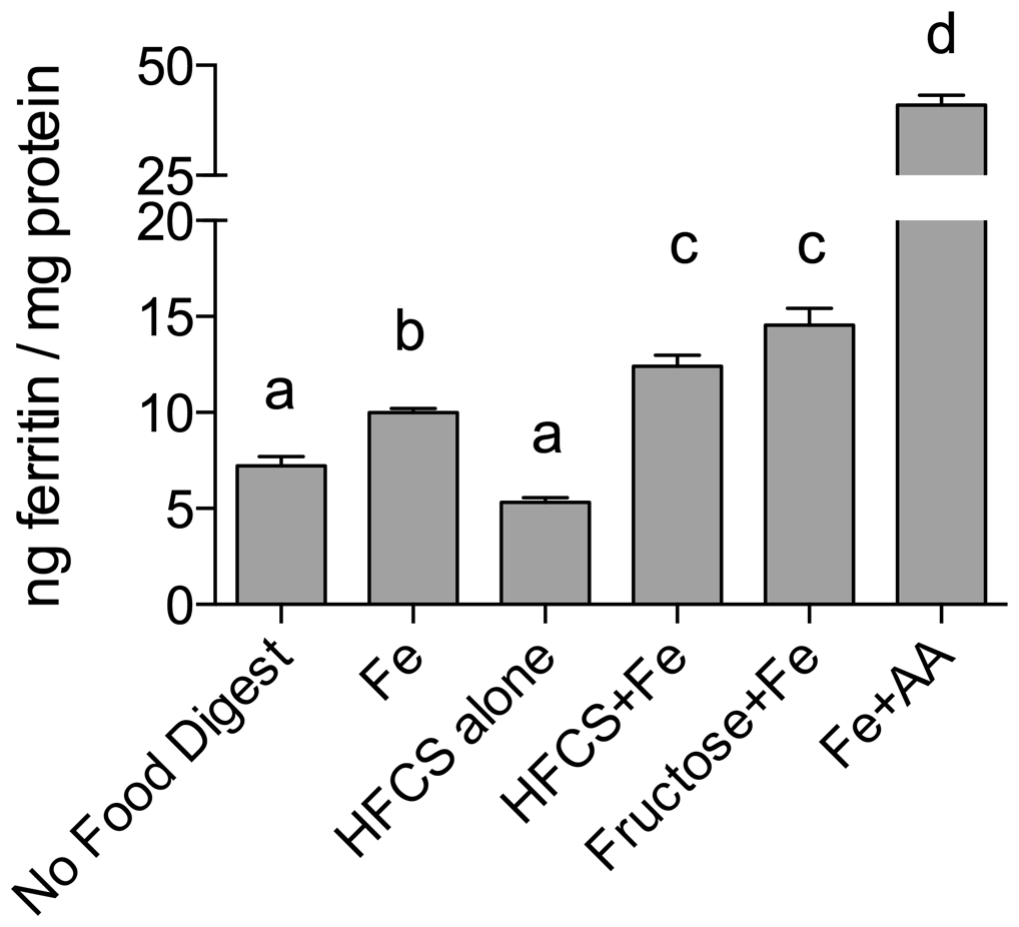
Effect of high-fructose corn syrup (HFCS) digests on iron-induced ferritin. Measurement of Caco-2 cell ferritin formation from digests of Fe and fructose (Fructose+Fe), or Fe and HFCS-55 (HFCS+Fe) at an iron:fructose ratio of ≈ 1:2000. Equal amounts of iron (25 µg) were combined with fructose solutions (1.0 mL) and subjected to the Caco-2 in vitro digestion process. Digests with HFCS alone and no added Fe (No Food Digest) were used as negative controls; digests with Fe alone (Fe) and Fe plus ascorbic acid (Fe + AA) were used as reference controls and positive controls, respectively. Values are means of data normalized to 10 ng of ferritin/mg protein in the reference control (Fe) ± SEM, n ≥ 18. Based on an ANOVA (p<0.0001) with Tukey’s multiple comparisons test post-hoc analysis done on an all-pairwise basis, bar values with no letters in common are significantly different (p ≤ 0.010).

The effect of sugars on iron bioavailability was assessed by incubating Caco-2 cells with digests of sugar solutions containing iron. Fructose increased iron-induced ferritin formation in Caco-2 cells by approximately 40% (Figure 1). In contrast, ferritin levels were not altered in cells exposed to iron plus either glucose or sucrose. In addition, incubation with digests of HFCS-55 increased iron-induced ferritin levels by approximately the same amount as fructose (Figure 2). Incubation with sugars alone (i.e. in the absence of iron) did not increase ferritin formation (data for glucose and sucrose not shown). Fructose levels in stock fructose solutions, and in stock HFCS-55 solutions after Chelex treatment, were on average 1 mol/L ± 0.01 (SEM) and 0.99 mol/L ± 0.04 (SEM), respectively.

### Effect of inhibitors of iron bioavailability on sugar-induced ferritin formation

**Figure 3:**
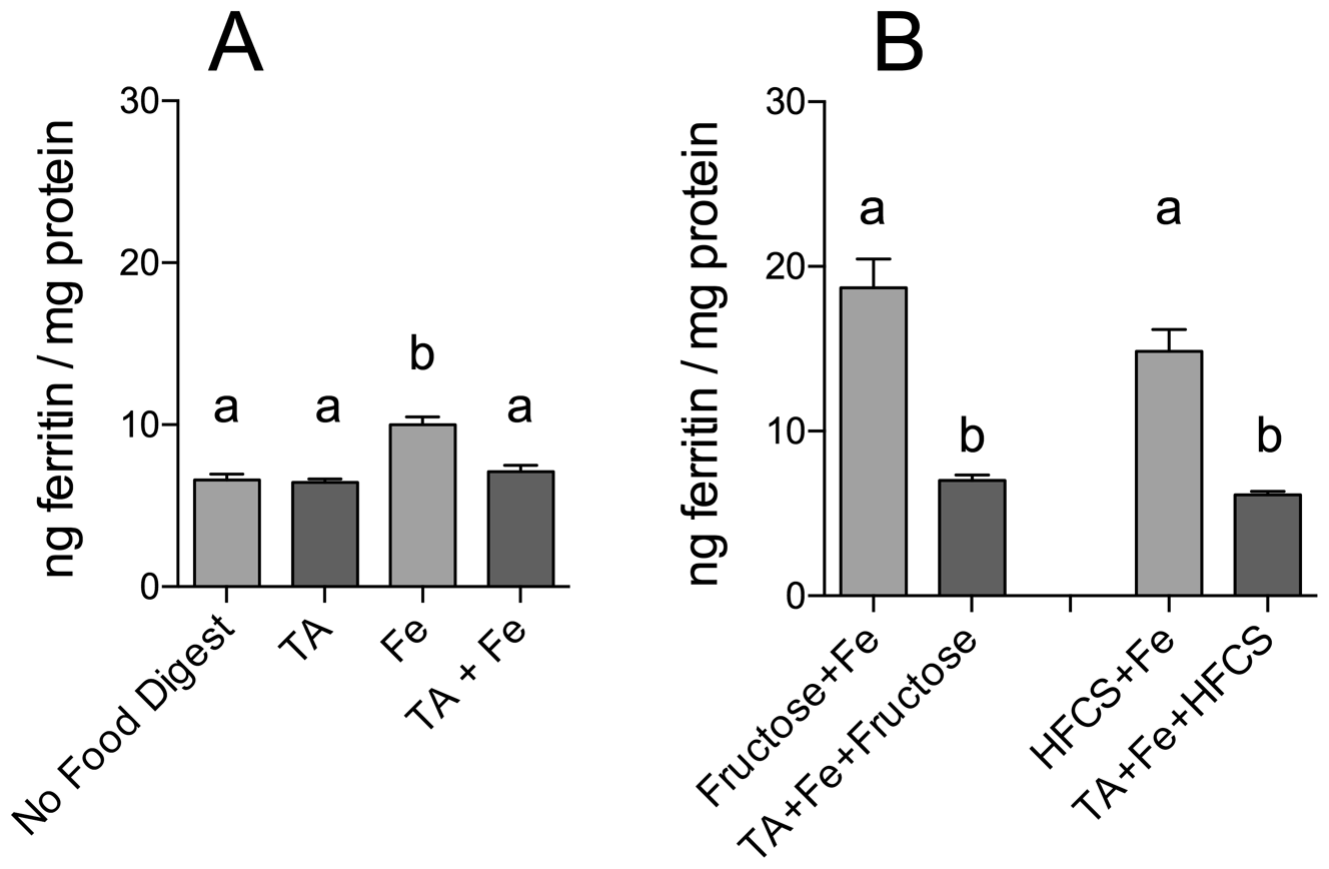
Effect of tannic acid (TA) and fructose, or TA and high-fructose corn syrup (HFCS), on iron-induced ferritin formation. Measurement of Caco-2 cell ferritin formation from digests of Fe and fructose (Fructose+Fe), or HFCS-55 (HFCS+Fe), at an iron:fructose ratio of ≈ 1:2000, plus tannic acid at a 1∶1 molar ratio of Fe:TA. Equal amounts of iron (25 µg) were combined with sugar solutions (1.0 mL) and TA and subjected to the Caco-2 in vitro digestion process. Digests without TA are shown with lighter shading and digests with TA added are shown with darker shading. Values are means of data normalized to 10 ng of ferritin/mg protein in the reference control (Fe) ± SEM, n(Fe+AA+TA) = 4, n(TA alone) = 6, all other n  =  18. Analysis of Figure 3A was based on a two-factor ANOVA (p<0.0001) with Tukey’s multiple comparisons test post-hoc analysis done on an all-pairwise basis, bar values with no letters in common are significantly different (p ≤ 0.010). Analysis of Figure 3B was based on a one-factor ANOVA (p<0.0001) with Tukey’s multiple comparisons test post-hoc analysis done on an all-pairwise basis, bar values with no letters in common are significantly different (p ≤ 0.010).

**Figure 4:**
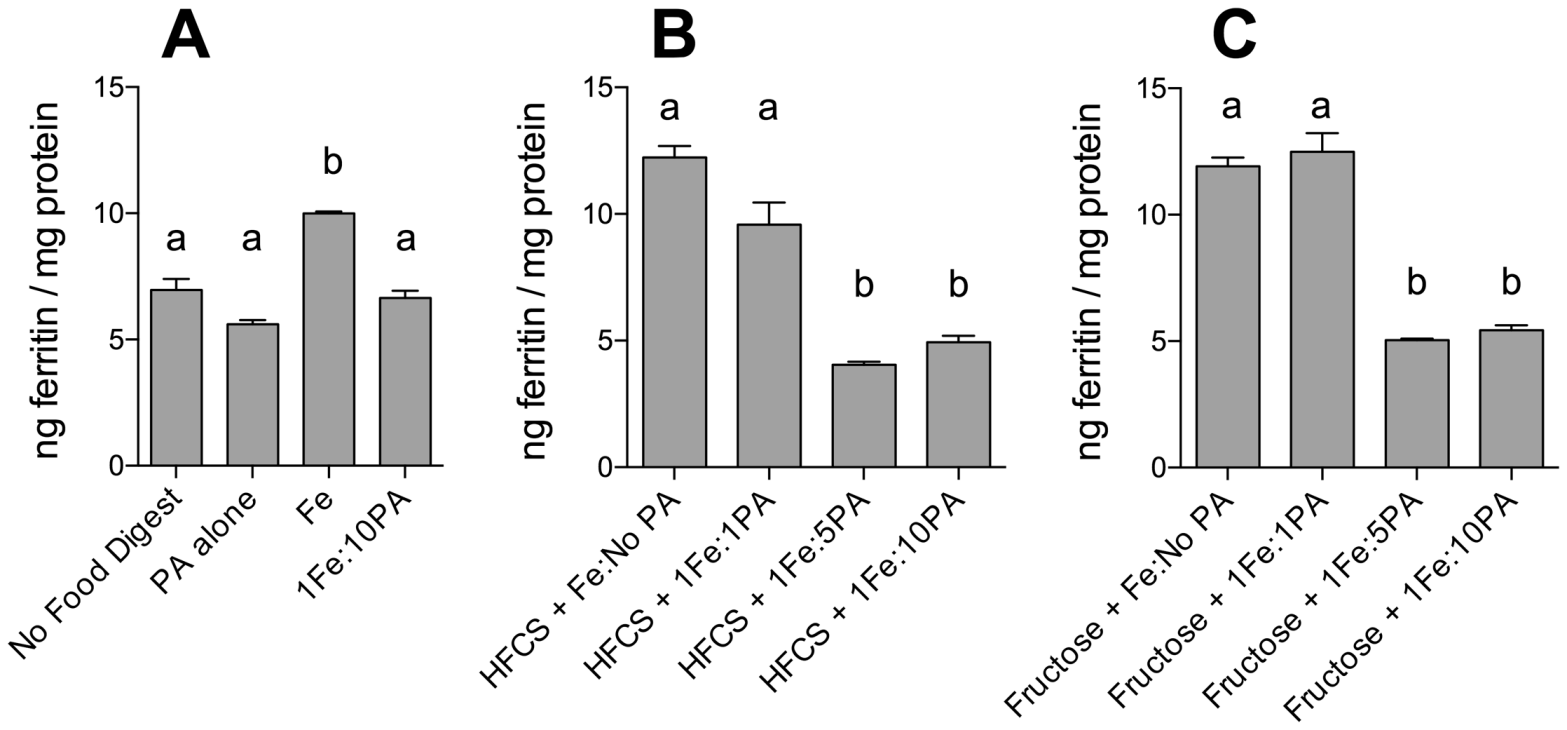
Effect of phytic acid (PA) and fructose, or high-fructose corn syrup (HFCS), on iron-induced ferritin formation. Measurement of Caco-2 cell ferritin formation from digests of Fe and fructose (Fructose+Fe), or HFCS-55 (HFCS+Fe), at an iron:fructose ratio of ≈ 1:2000, plus phytic acid at 1∶1, 1∶5 or 1∶10 Fe:PA molar ratios. Equal amounts of iron (25 µg) were combined with sugar solutions (1.0 mL) and PA and subjected to the Caco-2 in vitro digestion process. Digests of the above without the addition of PA are provided for reference. Digests with Fe alone (Fe), PA alone (PA Alone), and Fe plus PA (1Fe:10PA) were used as controls. Values are means of data normalized to 10 ng of ferritin/mg protein in the reference control (Fe) ± SEM, n(Fe+PA1:10) = 3, all other n ≥ 6. Analysis of Figure 4A was based on a two-factor ANOVA (p<0.0001) with Tukey’s multiple comparisons test post-hoc analysis done on an all-pairwise basis, bar values with no letters in common are significantly different (p ≤ 0.010). Analysis of Figures 4B and 4C was based on a one-factor ANOVA (p<0.0001) with Tukey’s multiple comparisons test post-hoc analysis done on an all-pairwise basis, bar values with no letters in common within Figure 4B are significantly different (p ≤ 0.010) and similarly bar values with no letters in common within Figure 4C are significantly different (p ≤ 0.010).

To determine whether known inhibitors of iron bioavailability could influence the enhancing effect of fructose and HFCS-55 on iron-induced ferritin formation, cells were incubated with either tannic acid (TA) or phytic acid (PA). Incubation with TA or PA alone did not alter basal cell ferritin levels (Figures 3 & 4). TA (1Fe:1TA molar ratio; Figure 3) and PA (1Fe:10PA molar ratio; Figure 4) both decreased iron bioavailability. Furthermore, addition of TA (Figure 3) or PA (Figure 4) to fructose- and HFCS-55-iron digests significantly decreased the sugar-iron-induced increase in ferritin formation to the level of “no food digests.”

### Effects of sugars on ferrous iron formation in vitro

**Figure 5:**
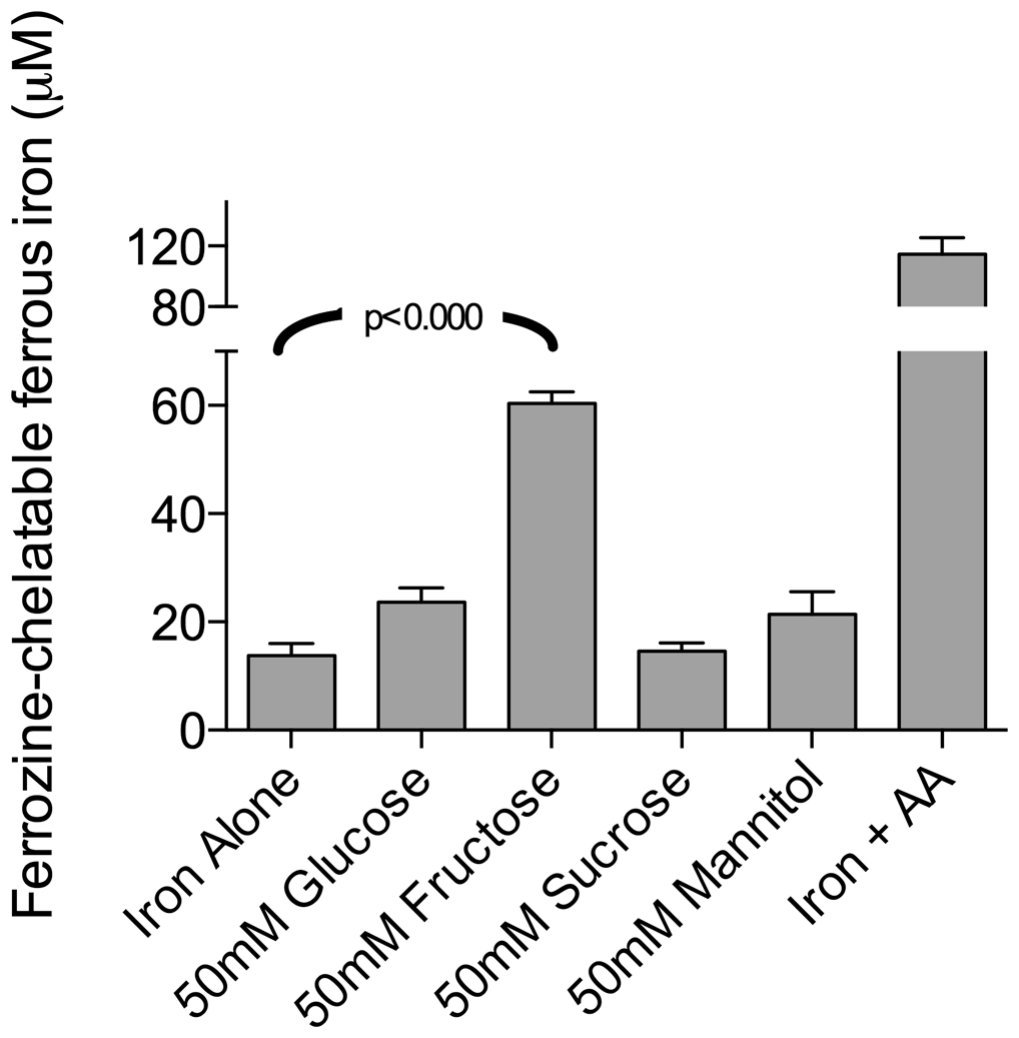
Carbohydrate effect on release of ferrozine-chelatable ferrous iron (Fe^2+^) in vitro. 50/L solutions of glucose, fructose, sucrose or mannitol were prepared with the addition of FeCl_3_ at a final concentration of 0.1 mmol/L. Solutions with iron alone and iron plus ascorbate were used as controls. Analysis for ferrozine-chelatable ferrous iron was performed after 2 hours incubation. Data in each column are presented as the mean ± SEM, n = 12 per group. Analysis was based on a one-factor ANOVA (p = 0.0001). Post-hoc analysis was done versus control. Fructose at a concentration of 50 mmol/L significantly increases ferrous iron levels in comparison to all other tested carbohydrate solutions; p < 0.0001, compared with 0.1 mmol/L FeCl_3_ alone.

It has been reported previously that sugars may have weak iron reducing and chelating activity [1], [25]. Therefore, to determine whether sugar solutions increased iron bioavailability via in vitro reduction of Fe^3+^ to Fe^2+^, we used the ferrozine assay that selectively detects ferrous iron. Fructose significantly increased ferrozine-chelatable ferrous iron levels by approximately 300% (Figure 5). There was no effect of glucose, sucrose or mannitol on Fe^2+^ formation. FeCl_3_ alone in MEM gave rise to the lowest levels of ferrozine-chelatable ferrous iron, with levels only 22% of those formed in the presence of fructose.

### Effect of fructose on HepG2 cell ferritin formation

**Figure 6:**
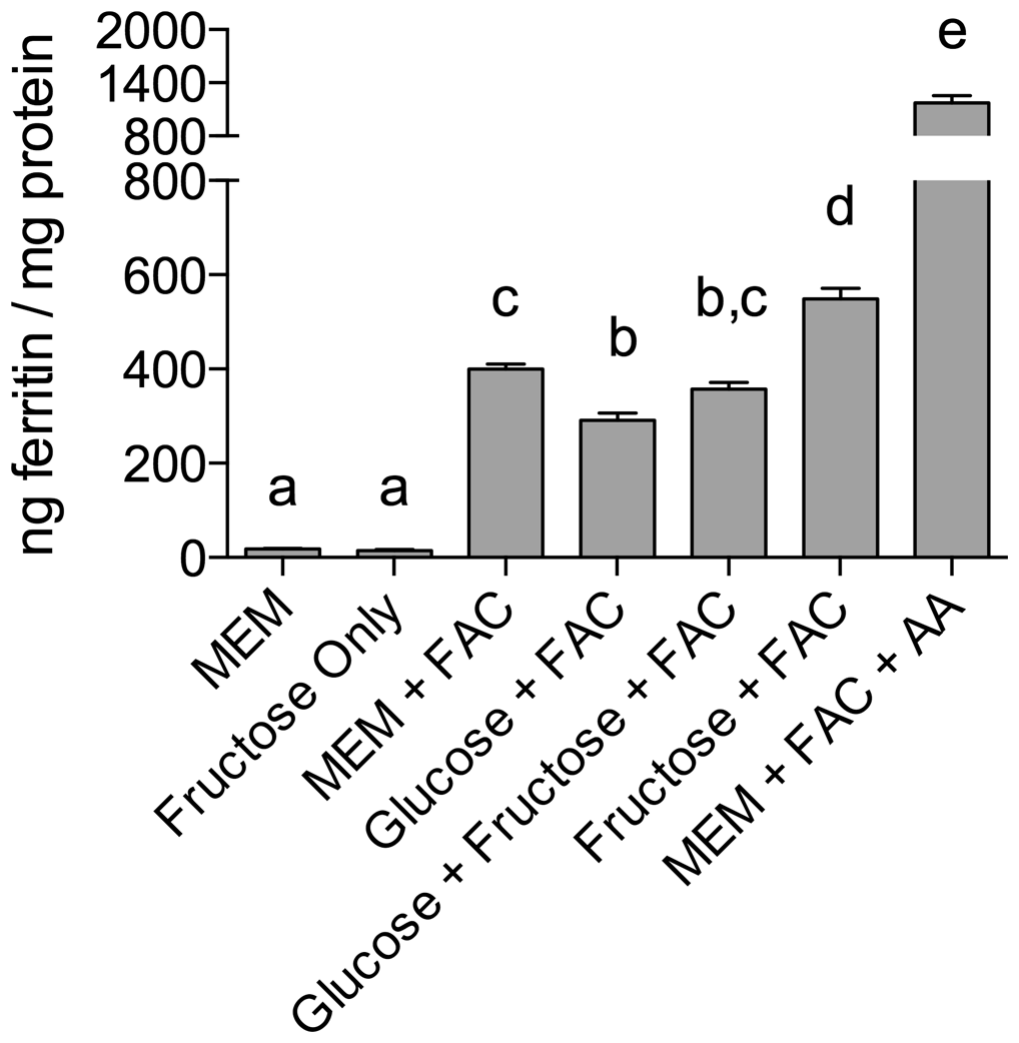
HepG2 iron-induced ferritin in response to carbohydrate treatments. Measurement of HepG2 cell ferritin formation following treatment for 24 µmol/L ferric ammonium citrate (FAC) and one of the following: 15 mmol/L glucose (Glucose+FAC); 15 mmol/L glucose and 15 mmol/L fructose (Fructose+Glucose+FAC); 15 mmol/L fructose (Fructose+FAC). Cells treated with MEM alone (MEM), or fructose alone (Fructose Only), without the addition of FAC, were used as negative controls. Cells treated with 0.1 mmol/L ascorbate and 1 µmol/L FAC were used as positive controls. Values are means of data normalized to 400 ng of ferritin/mg protein in the reference control (MEM+FAC) ± SEM, n(MEM) = 4, n(Fructose Only) = 6, all other n ≥ 12. Based on an ANOVA (p<0.0001) with Tukey’s multiple comparisons test post-hoc analysis done on an all-pairwise basis, bar values with no letters in common are significantly different (p ≤ 0.010).

**Figure 7:**
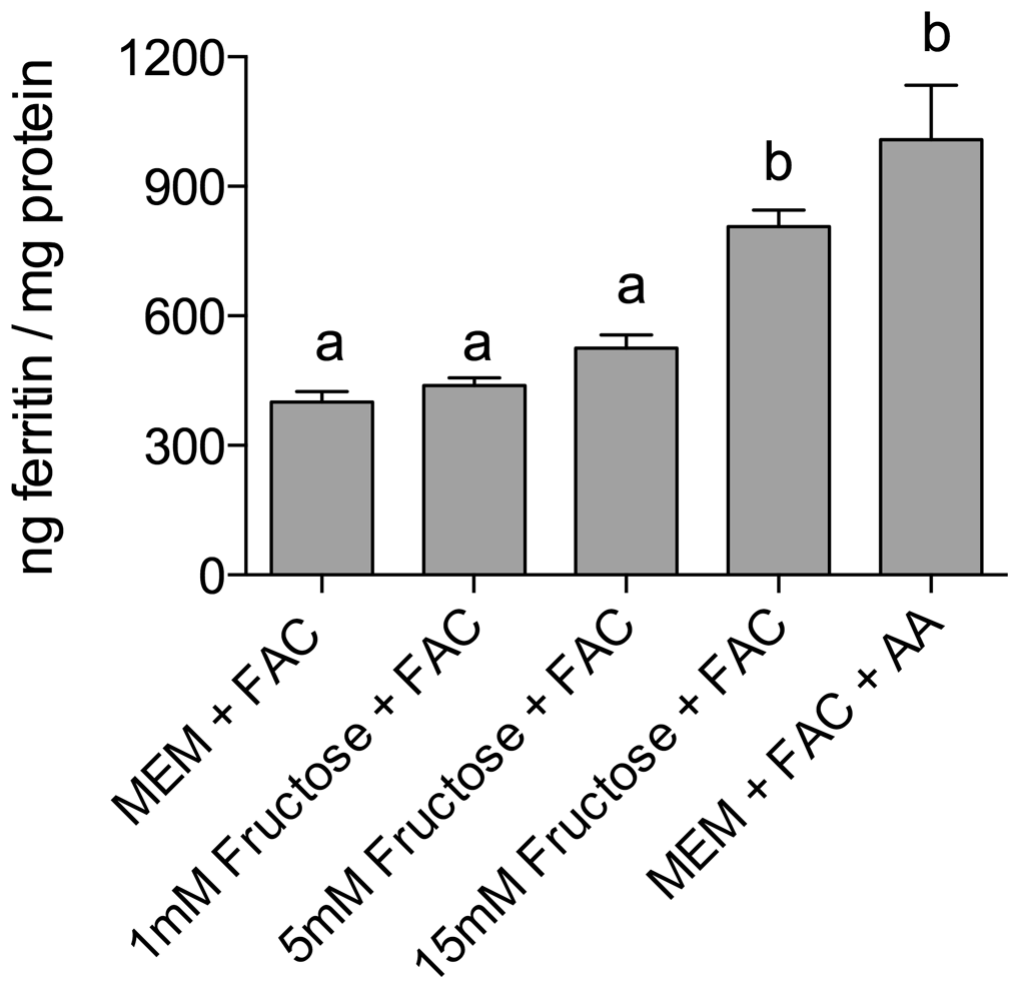
Fructose dose response on HepG2 cell iron-induced ferritin. Measurement of HepG2 cell ferritin formation following treatment for 24 µmol/L FAC and one of the following: 1 mmol/L fructose (1 mM Fruc+FAC); 5 mmol/L fructose (5 mM Fruc + FAC); 15 mmol/L fructose (15 mM Fruc + FAC), to determine dose response of HepG2 cell ferritin relative to fructose concentration. Values are means of data normalized to 400 ng of ferritin/mg protein in the reference control (MEM+FAC) ± SEM, n ≥ 6. Based on an ANOVA (p<0.0001) with Tukey’s multiple comparisons test post-hoc analysis done on an all-pairwise basis, bar values with no letters in common are significantly different (p ≤ 0.010).

To determine whether fructose or glucose might also influence hepatic iron-induced ferritin formation HepG2 cells were exposed to increasing concentrations of fructose, or glucose, and iron. In the presence of iron, liver ferritin levels were unaffected by co-addition of glucose; however, fructose increased iron-induced HepG2 cell ferritin by approximately 35% - maximal ferritin formation was observed with 15 mmol/L fructose (Figures 6 &7). There was no effect of fructose alone on ferritin formation (Figure 6).

## Discussion

Non-heme iron bioavailability is influenced by many dietary factors. This study suggests that fructose increases iron bioavailability in our in vitro cell models of the gut and liver. These results are consistent with previous work in rodent models in which iron-fructose solutions increased both gut iron absorption [18] and liver iron deposition [47].

Recent human intervention trials looking at the effects of fructose on iron uptake are lacking, but there have been several epidemiological studies that analyzed fruit intake and iron status. Fruit is a dietary source of fructose; observational studies of fruit as a modifier of iron status have yielded conflicting results. Milward et al carried out a study in subjects with hereditary hemochromatosis (HH), which found that non-citrus fruit intake was protective against iron overload [48]. In contrast, Fleming et al found that fruit intake was associated with an increased risk for elevated iron stores in the Elderly Framingham Heart Study cohort [49]. The different results are most likely secondary to several factors. The study by Milward et al differentiated between citrus fruits (which are rich sources of citric and ascorbic acid, known enhancers of iron uptake), and non-citrus fruit; Fleming et al did not differentiate between fruit types. In addition, Milward et al only studied subjects with HH, whereas HH was one of the exclusion criteria for the Framingham study. Another possible confounding factor is that fruits have varying levels of phytates and polyphenols; the results of our study suggest that fructose in fruit would not alter iron bioavailability as both phytate and the polyphenol tannic acid inhibited the effects of fructose on iron bioavailability.

In Western diets and increasingly worldwide, however, the major source of fructose in the diet is not fruit, but sucrose and high-fructose corn syrup, and in particular HFCS-55 [27], [50]. In the United States of America HFCS represents 15–20% of total energy intake, the majority coming from sugar sweetened beverages (SSB) [51]. Here, we investigated the effect of HFCS-55 on iron bioavailability, and demonstrated increased ferritin formation in the Caco-2 cell system. Few studies have investigated the effects of sugars from SSB on iron bioavailability. A small study by Hallberg et al found that a low pH cola drink increased iron absorption; however, this was attributed to the low pH of the beverage and data on the carbohydrate composition of the beverage is unavailable [52]. A more recent study, which specifically looked at the effect of beverage carbohydrate on iron bioavailability by comparing regular cola with artificially sweetened diet cola, found no effect on iron absorption from either source [53]. However, cola drinks contain between 0.5 – 0.7 mmol/L caffeine [54], a polyphenol shown to decrease non-heme iron bioavailability in multiple studies and thus a possible confounding factor when assessing SSB sugar effects on iron bioavailability [55], [56]. In our study HFCS-55, used at a concentration comparable to that found in sweetened beverages, significantly increased iron bioavailability and the effect was comparable to that observed with fructose.

The other sugars tested in this study did not influence iron bioavailability and this is consistent with previous work showing no effects of sucrose or glucose on iron absorption [18]. One might have predicted that the fructose released from digestion of sucrose would increase iron uptake. However, there are suggestions in the literature that sucrose-derived fructose is taken up by the enterocyte immediately upon hydrolysis; it would therefore not be available to interact with iron, in comparison with HFCS which upon ingestion yields free fructose monomers in the intestinal lumen [57].

It has been proposed that fructose increases iron bioavailability by increasing ferrous iron formation [25]. Our observation that fructose significantly increased ferrozine-chelatable ferrous iron levels is consistent with this mechanism. Fructose is a reducing sugar giving positive tests for both Benedicts and Fehlings reagents; in solution it exists primarily in the furanose form but is in equilibrium with the straight chain and pyranose forms [58]. Interestingly, in basic solution fructose is a stronger reducing agent than aldoses such as glucose [59]. Sucrose is not a reducing sugar because neither of its carbonyl groups are available to participate in redox reactions [60].

Work by Stitt et al looking at sugar effects on iron bioavailability analyzed iron levels in the liver, as well as gut iron uptake; iron co-administered with fructose resulted in both increased iron absorption and liver iron deposits [47]. In addition, a recent study using mice found that a high-fructose/high-fat diet increased iron liver levels [61]. These observations are consistent with our findings that HepG2 ferritin levels increased in cells treated with iron and fructose. Data on human blood fructose levels are limited, and there is even less information on human portal vein fructose concentrations to which the liver would be exposed. However, a recent study using an enzyme-based assay, validated by gas chromatography-mass spectroscopy, reported circulating serum post-prandial fructose levels up to 16 mmol/L [62]; if these values are correct then portal vein fructose levels would be predicted to be in the order of 27 - 53 mmol/L as the liver extracts 40–70% of portal vein fructose [63]. Older studies in animals, and several small studies in humans, have reported portal vein fructose levels varying from 1 – 2.2 mmol/L [63]–[67]. The fructose concentrations used in our studies lie somewhere in the middle of these two extremes of possible post-prandial portal circulation levels.

A number of studies have used either high fructose or HFCS diets to augment the effects of a high fat diet on liver metabolism, in order to study the development of fatty liver. Given that liver iron loading is a common feature in human fatty liver disease [8], the observations that fructose and HFCS increased iron uptake in our in vitro models of both gut and liver suggests that these carbohydrates may have an important pathological role.

Under normal conditions the majority of circulating iron is bound to the plasma iron transport protein transferrin [68], however blood levels of 0 – 1μmol/L non-transferrin bound iron (NTBI) may occur post-prandially [69]. Furthermore, NTBI levels may reach 1 – 20 µmol/L in iron overload disorders as well as in other chronic diseases such as liver cirrhosis [70]. NTBI is a heterogeneous mix of compounds; studies suggest that the main form of plasma NTBI is iron(III)citrate [71], for this reason FAC is an appropriate model for plasma NTBI. Interestingly, hepatocyte uptake of NTBI, as opposed to transferrin iron uptake, does not appear to be inhibited by increasing levels of liver iron [72], [73]; fructose mediated liver NTBI uptake may thus escape regulation.

In conclusion, we have shown that fructose and HFCS-55 increase iron bioavailability in human intestinal epithelial cells and, furthermore, that fructose increases iron-induced hepatic ferritin levels. Given that substantial amounts of these carbohydrates are present in the modern diet, and also their use in experimental models, these effects may be important in the context of iron homeostasis. Further studies are warranted to examine if these in vitro effects translate into (patho)physiologically relevant changes in animal and human iron status.
